# A Case of Visceral Leishmaniasis in an Immunocompetent Adult

**DOI:** 10.7759/cureus.101798

**Published:** 2026-01-18

**Authors:** Joana Coelho, Ana Silva, Lúcia Jardim, Juliana Carneiro, Marlene Louro

**Affiliations:** 1 Internal Medicine, Unidade Local de Saúde da Cova da Beira, Covilhã, PRT

**Keywords:** fever of unknown origin, immunocompetent adult, mediterranean endemic disease, pancytopenia, serological diagnosis, splenomegaly, visceral leishmaniasis

## Abstract

Visceral leishmaniasis is a rare parasitic infection in immunocompetent individuals, being more frequent in children and immunocompromised individuals. Despite being endemic in several regions of the world, including Portugal, it continues to be underdiagnosed. The most characteristic clinical manifestations include constitutional symptoms, pancytopenia, polyclonal hypergammaglobulinemia, and splenomegaly. Direct identification of the parasite is the preferred diagnostic method; however, this approach is not always feasible. Consequently, serological testing becomes essential, particularly in immunocompetent individuals when supported by a compatible clinical, laboratory, and epidemiological context. We present the case of a 72-year-old woman residing in a rural area of Portugal who developed an insidious course of constitutional symptoms accompanied by pancytopenia, polyclonal hypergammaglobulinemia, elevated erythrocyte sedimentation rate, and splenomegaly. After an extensive diagnostic evaluation and exclusion of multiple infectious, hematologic, autoimmune, and neoplastic disorders, serological testing for leishmaniasis returned positive results, despite the inability to directly visualize the parasite in the bone marrow aspirate. Liposomal amphotericin B was initiated; however, the patient developed urinary septic shock and died. This case highlights the importance of considering VL in immunocompetent adults with a suggestive clinical, laboratory, and epidemiological context. It also emphasizes the diagnostic complexity of the disease and the value of serological methods in establishing the diagnosis, enabling early recognition and helping to avoid delays in management.

## Introduction

Leishmaniasis is an anthropozoonotic disease caused by protozoa of the genus *Leishmania* and is transmitted through the bite of infected female sandflies of the genera *Phlebotomus* and *Lutzomyia* [1]. It encompasses cutaneous, mucocutaneous, and visceral forms. Visceral leishmaniasis (VL) is primarily caused by *Leishmania donovani* and *Leishmania infantum* [2].

VL is endemic in approximately 100 countries, with an estimated 30,000 new cases reported annually in these regions and about 700 new cases per year in Europe [3]. In Portugal, between 2010 and 2020, 221 cases were identified in public hospitals, although the true number may be higher due to underreporting [3].

The infection may range from asymptomatic to severe and can present in a more insidious manner, with prolonged fever, splenomegaly with or without hepatomegaly, pancytopenia, and constitutional symptoms [1,2]. Occurrence in immunocompetent adults is rare, being more frequently observed in immunocompromised individuals [1,2]. The diagnosis can be challenging, as direct identification of the parasite is not always feasible; however, serological tests demonstrate high sensitivity in immunocompetent patients when interpreted in conjunction with the clinical presentation and epidemiological context [2,4].

With regard to treatment, therapeutic decision-making generally does not require species identification, as it is based on disease severity, the presence of comorbidities or coinfections, such as human immunodeficiency virus (HIV) infection, and regional variability in drug susceptibility [2,5]. Through this article, we aim to emphasize that, despite the relative rarity of VL in developed countries, its diagnosis should not be overlooked.

## Case presentation

We present the case of a 72-year-old Caucasian woman, born in Portugal and residing in a rural area of the Serra da Estrela region. Her past medical history was significant for arterial hypertension, type 2 diabetes mellitus, dyslipidemia, and obesity, with no other relevant comorbidities. She was receiving medical therapy appropriate for the aforementioned conditions. There was no significant family medical history. Her immunizations were up to date according to the Portuguese National Immunization Program. The patient owned two dogs that were vaccinated and reported no contact with other animals. She lived in a house supplied with public water, did not consume unpasteurized products, had not traveled to areas of epidemiological relevance, and denied high-risk sexual behaviors.

She was evaluated in the Internal Medicine outpatient clinic for fatigue, asthenia, and unintentional weight loss (approximately 5% of body weight over six months), with symptoms evolving over several months and progressively worsening. Additionally, during the preceding month, she reported an irregular fever pattern, predominantly in the afternoon (maximum temperature of 38.9°C), which responded to 1 g of acetaminophen.

On physical examination, she appeared cachectic, with pale mucous membranes and splenomegaly, with the splenic edge palpable 3 cm below the costal margin. No other significant abnormalities were noted. Laboratory findings are summarized in Table 1, with notable pancytopenia, elevated erythrocyte sedimentation rate, mild cytocolestasis, hypoalbuminemia, and serum protein electrophoresis revealing polyclonal hypergammaglobulinemia, with no evidence of monoclonality on immunofixation. The patient was admitted to the Infectious Diseases Department for further diagnostic evaluation.

**Table 1:** Initial laboratory evaluation AST: aspartate aminotransferase; ALT: alanine aminotransferase; LDH: lactate dehydrogenase; GGT: gamma-glutamyl transferase; FA: alkaline phosphatase; CRP: C-reactive protein; ESR: erythrocyte sedimentation rate

Parameter	Initial values	Reference range
Leukocytes	2600/µL	4000-10000/µL
Neutrophils	1200/µL	1500-8000/µL
Lymphocytes	1100/µL	800-4000/µL
Monocytes	200/µL	0-1200 /µL
Eosinophils	0/µL	0-700 /µL
Hemoglobin	8.1 g/dL	11.5-16.0 g/dL
Hematocrit	25.4 %	34.7-46.0 %
Platelets	12000/µL	150-450/µL
ESR	80 mm/H	0-20 mm/H
Iron	70.4 µg/dL	60.0-180.0
Transferrin saturation	76.8 %	15.0-45.0
Ferritin	1282 ng/mL	13.0-150.0
Urea	75 mg/dL	17-48 mg/dL
Creatinine	1.55 mg/dL	0.50-0.90 mg/dL
AST	48 U/L	0-32 U/L
ALT	55 U/L	0-31 U/L
LDH	232 U/L	125-220 U/L
GGT	149 U/L	7-139 U/L
ALP	301 U/L	35-104 U/L
Total bilirubin	2.0 mg/dL	0,00-1.10 mg/dL
Total proteins	6.9 g/dL	6.4-8.3 g/dL
Albumin	2.4 g/dL	3.4-4.8 g/dL
CRP	1.17 mg/dL	0.00-0.50 mg/dL

Complementary investigations revealed negative results for Cytomegalovirus, Epstein-Barr virus, human immunodeficiency virus (HIV), hepatitis B and C viruses, *Brucella*, *Coxiella*, *Borrelia*, *Rickettsia*, and interferon-gamma release assay (IGRA). Blood cultures were also negative.

**Figure 1:**
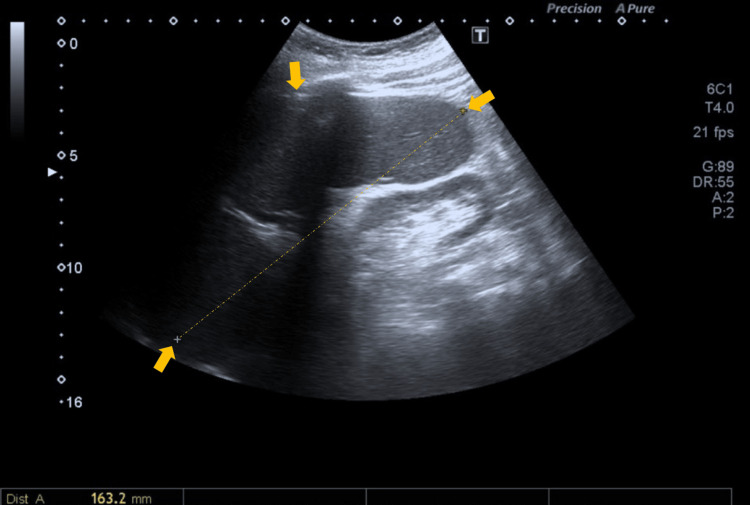
Abdominal ultrasound revealing splenomegaly The yellow arrows indicate splenomegaly, and the line indicates the spleen length

Examination for microorganisms and parasites on peripheral blood smear and thick blood drop was negative. Bone marrow aspirate, including morphological evaluation and staining for microorganisms and parasites, showed no abnormalities, with no evidence of neoplastic or granulomatous infiltration. Autoimmune screening was negative. Abdominal ultrasound demonstrated only splenomegaly measuring 16 cm (Figure 1).

Serological testing for Leishmania was performed using indirect immunofluorescence assay (IFA) and enzyme-linked immunosorbent assay (ELISA). Based on the clinical and laboratory findings, and after exclusion of other infectious, hematologic, autoimmune, and malignant causes (such as brucellosis, tuberculosis, malaria, histoplasmosis, schistosomiasis, lymphomas, leukemias, myelodysplastic syndromes, systemic lupus erythematosus, and hemophagocytic lymphohistiocytosis/macrophage activation syndrome), a diagnosis of VL was established. The patient completed seven days of liposomal amphotericin B at a dose of 3 mg/kg/day. However, she subsequently developed septic shock of urinary origin due to *Klebsiella pneumoniae *and, despite all directed therapeutic interventions (such as antibiotic therapy, fluid therapy, and vasopressor support), she deteriorated and died on the 11th day of treatment.

## Discussion

VL is considered an underdiagnosed condition and is classified by the World Health Organization as a neglected disease [6]. It is rare in immunocompetent individuals in European countries, including Portugal. Traditionally, VL has been described in children and immunocompromised patients, particularly those with HIV infection or receiving immunosuppressive therapy [2,6]. In Portugal, VL caused by *Leishmania infantum* is considered endemic [7], with domestic dogs serving as the primary reservoir [8].

The present case involves an elderly immunocompetent patient, illustrating that VL continues to occur outside the traditional risk groups, highlighting the importance of including it in the differential diagnosis. The patient’s epidemiological context is particularly relevant, as Portugal is an endemic country, and the Serra da Estrela region has been reported to have a considerable incidence of VL, coinciding with a higher prevalence of canine leishmaniasis, as demonstrated by the study by Rocha et al. [3].

The presentation of constitutional symptoms, pancytopenia, polyclonal hypergammaglobulinemia, and splenomegaly is characteristic of VL; however, it is highly nonspecific and may occur in a wide range of infectious, hematologic, and autoimmune conditions [2,9]. Therefore, establishing the diagnosis requires an individualized, systematic approach in accordance with current recommendations.

Definitive diagnosis is made by direct demonstration of the parasite in bone marrow, spleen, or blood samples [1,2]. However, this is not always feasible. Comparative studies indicate that serological tests, including indirect IFA, ELISA, direct agglutination test, and the rK39 rapid immunochromatographic test, play a critical role, particularly in such cases [10-12]. According to major international guidelines and reviews, in immunocompetent adults, diagnosis can be established based on a typical clinical presentation and positive serological tests in a compatible epidemiological context [2,4,13,14]. It should be noted, however, that serological tests have low sensitivity in immunocompromised patients [2,4,10,11,13,14]. In the present case, the patient exhibited the characteristic clinical features described above, resided in an area with confirmed circulation of both the parasite and its vector, and had positive specific serological tests. Taken together, these findings support the diagnosis of VL, even in the absence of parasite isolation from the bone marrow. Furthermore, as previously mentioned, the main differential diagnoses were excluded, including viral, bacterial, and parasitic infections, malignant hematologic diseases, and autoimmune or inflammatory conditions [1,2].

Thus, this case highlights the relevance of considering VL as a potential diagnosis in immunocompetent adults, even in regions where incidence may be lower. It is important to note that infection in immunocompetent individuals can result from the interaction between parasite, host, and environmental factors, associated with a less effective cellular immune response, which favors the persistence and spread of the parasite, even in the absence of obvious immunodeficiency [15]. This underlines the importance of serological testing, particularly in immunocompetent patients and when direct demonstration of the parasite is not feasible, a scenario reported in multiple studies in the literature.

Another important point, also discussed in the literature, is the immune dysfunction associated with VL, which predisposes patients to severe and potentially fatal bacterial infections [2], as occurred in this case. This can happen even when treatment for VL is initiated according to current recommendations [5,9]. Consequently, close monitoring and careful clinical management are required.

Finally, it is important to acknowledge that this is a single case report and that molecular studies for more precise parasitological characterization, which could contribute to epidemiological surveillance, were not performed. Nevertheless, these limitations do not diminish the illustrative value of the case, which contributes to raising awareness of the diagnosis and supports the continuation of surveillance and epidemiological control programs in endemic areas.

## Conclusions

In summary, VL should be considered in the differential diagnosis of constitutional syndromes with pancytopenia in immunocompetent individuals residing in endemic areas. This case underscores the epidemiological relevance, the need for a high index of clinical suspicion, and the diagnostic complexity of the disease. It highlights the crucial role of early incorporation of serological methods in the diagnostic process, particularly in immunocompetent patients and when direct demonstration of the parasite is not feasible. Furthermore, it reinforces the ongoing need for epidemiological surveillance in at-risk regions.
